# Bladder Dysfunction as One of the Overlooked Comorbidities in Adults with Diabetes

**DOI:** 10.31662/jmaj.2026-0099

**Published:** 2026-07-24

**Authors:** Atsushi Araki

**Affiliations:** 1 Department of Diabetes, Endocrinology, and Metabolism, Tokyo Metropolitan Institute for Geriatrics and Gerontology, Tokyo, Japan

**Keywords:** diabetes mellitus, bladder dysfunction, urinary incontinence, nocturia, quality of life

In this issue of the *JMA Journal*, Chawla et al. ^(1)^ reported that, in a cross-sectional study, bladder dysfunction was associated with poor sleep quality in outpatients with type 2 diabetes. Both urinary incontinence and poor sleep quality were independently related to a lower quality of life, as assessed using the Short Form-36 ^(1)^. This study emphasizes the importance of evaluating and treating bladder function and lower urinary tract symptoms (LUTS) as overlooked complications in the clinical management of diabetes mellitus.

LUTS include storage symptoms, voiding symptoms, and postvoiding symptoms. The prevalence of LUTS is increasing in Japan as its population ages. A study of 6,210 Japanese participants found that the overall prevalence of LUTS was 82.5% among those aged 40 and older, increasing with age ^(2)^.

Diabetes is also associated with impaired bladder dysfunction. The prevalence of LUTS is estimated to occur in 50%-80% of patients with diabetes ^(3)^. The early phase of bladder dysfunction is manifested by storage problems (such as urgency and urge incontinence), while voiding problems (inability of the bladder to empty causing high postvoid residual and overflow incontinence) occur in the late phase. LUTS are often manifested as a combination of storage and voiding bladder problems. Women with diabetes have a threefold increased prevalence of urge incontinence and twice the prevalence of stress incontinence compared with women without diabetes ^(4)^. A prospective study demonstrated that women with diabetes were associated with incidence of any incontinence and severe incontinence by 1.2- to 1.4-fold ^(5)^. Patients with type 2 diabetes also have more symptoms of overactive bladder, such as urgency and nocturia. The prevalence of mild nocturia (one episode) and moderate to severe nocturia (two or more episodes) among 762 Japanese patients with type 2 diabetes was 39.5% and 41.9%, respectively ^(6)^.

Age, hyperglycemia, diabetic peripheral or autonomic neuropathy, and obesity have been reported to be associated with LUTS in adults with type 1 or type 2 diabetes ^(7), (8), (9)^. Chawla et al. ^(1)^ also reported an association between bladder dysfunction and poor sleep quality. Bladder dysfunction and sleep disorders are geriatric syndromes, as are frailty, falls, depression, and cognitive impairment. These syndromes have common mechanisms that potentially involve aging processes, including insulin resistance, inflammation, and oxidative stress. These geriatric syndromes lead to a reduced quality of life and decreased healthy life expectancy.

Therefore, it is important to recognize and treat bladder dysfunction as a comorbidity that is often overlooked in adults with diabetes. Comprehensive treatment involving optimal blood glucose control, weight reduction, muscle-strengthening exercises (including those for the pelvic floor), nutrition, medical treatment, and social participation or support may be necessary for managing these geriatric syndromes. Avoiding smoking, caffeine, alcohol, and excessive water intake is also important.

Medical treatment for voiding dysfunction can include α1-adrenoceptor antagonists for both sexes or phosphodiesterase type 5 inhibitors for men. Muscarinic receptor antagonists have been used to treat overactive bladder with storage dysfunction. However, β3-adrenoceptor agonists have recently been shown to be more effective and safer due to their lack of anticholinergic effects. In Japan, desmopressin has become the first-line drug for treating nocturia with nocturnal polyuria in men. It is necessary to establish a clinical framework for the evaluation and treatment of bladder dysfunction in patients with diabetes that involves urologists and other multidisciplinary professionals.

## Article Information

### Conflicts of Interest

Araki A has received speaker honoraria from Novo Nordisk Pharma Ltd.
